# KHSRP-bound small nucleolar RNAs associate with promotion of cell invasiveness and metastasis of pancreatic cancer

**DOI:** 10.18632/oncotarget.27413

**Published:** 2020-01-14

**Authors:** Keisuke Taniuchi, Mitsunari Ogasawara

**Affiliations:** ^1^Department of Gastroenterology and Hepatology, Kochi Medical School, Kochi University, Nankoku, Kochi 783-8505, Japan; ^2^Department of Endoscopic Diagnostics and Therapeutics, Kochi Medical School, Kochi University, Nankoku, Kochi 783-8505, Japan

**Keywords:** RNA-binding protein, small nucleolar RNA, pancreatic cancer, cell invasion, metastasis

## Abstract

KH-type splicing regulatory protein (KHSRP) is an RNA-binding protein implicated in a variety of cellular processes, including splicing in the nucleus and mRNA localization and degradation in the cytoplasm. The present study reports that KHSRP promotes invasiveness and metastasis of pancreatic cancer cells. KHSRP was localized in the nucleus and cell protrusions of pancreatic cancer cell lines. Suppression of KHSRP by small interfering RNA decreased the number of cell protrusions and inhibited invasiveness and metastasis of pancreatic cancer cells. KHSRP was localized in cytoplasmic RNA granules in pancreatic cancer cells, and RNA immunoprecipitation-sequencing analysis showed that the majority of enriched RNAs that immunoprecipitated with KHSRP were small nucleolar RNAs (snoRNAs). Specific KHSRP-bound snoRNAs, *SNORA18* and *SNORA22*, associated with formation of cell protrusions. Consequently, *SNORA18* and *SNORA22* contributed to cell invasiveness and tumor metastasis. Our results provide insight into the link between KHSRP-bound snoRNAs and invasiveness and metastasis of pancreatic cancers. New therapies that prevent binding of KHSRP with specific snoRNAs may hold significant clinical promise.

## INTRODUCTION

RNA binding proteins play roles in RNA maturation and trafficking of RNAs, and localization of mRNAs is accomplished with intracellular accumulation of corresponding proteins [1]. KH-type splicing regulatory protein (KHSRP) is a single-stranded nucleic acid-binding protein that contains four contiguous K homology (KH) motifs that recognize the AU-rich element (ARE) of mRNAs [2] and functions in alternative pre-mRNA splicing, editing, localization, and degradation of mRNAs [3]. KHSRP phosphorylated by p38 increases binding to ARE-containing transcripts and inhibits rapid decay of target transcripts, resulting in conversion of myoblasts into differentiated myocytes [4]. KHSRP promotes decay of *β-catenin* mRNA and is inactivated by phosphatidylinositol 3-kinase (PI3K) signaling [5]. These results suggest that KHSRP is involved in differentiation, cell-cell contact, and cell migration through post-transcriptional regulation of its target transcripts. KHSRP also serves as a component of both Drosha and Dicer complexes and regulates biogenesis of a subset of microRNAs (miRNAs) [6]. This mechanism is required for post-translational regulation of target mRNAs that affect cell proliferation, apoptosis, and differentiation [6]. The functional roles of KHSRP in cell proliferation, invasiveness, and metastasis in cancer cells are currently unknown.

KHSRP is located primarily in the nucleus [7], where it acts as a splicing factor and forms part of the perinucleolar structure [8]. KHSRP is also present in cytoplasmic granules that function in RNA trafficking [9, 10], and KHSRP contributes to mRNA localization in the cytoplasm [11]. KHSRP binds to a localization element within the *β-actin* mRNA and has a role in cytoplasmic localization of *β-actin* mRNA to cell protrusions of chicken fibroblasts and neurite growth cones of developing neurons [12]. Localized translation of *β-actin* mRNA induces polarized migration of chicken fibroblasts [13]. Thus, it is possible that cytoplasmic RNA granules package KHSRP, and its target transcripts play a role in mRNA trafficking towards distal regions of the cell and regulation of localized protein synthesis.

Here, we found that KHSRP and its target small nucleolar RNAs (snoRNAs) were packaged into cytoplasmic RNA granules of pancreatic ductal adenocarcinoma (PDAC) cells. Further investigation revealed that KHSRP-bound snoRNAs influenced formation of cell protrusions and thereby increased invasive and metastatic properties of PDAC cells.

## RESULTS

### Intracellular localization of KHSRP in PDAC cells

**Figure 1:**
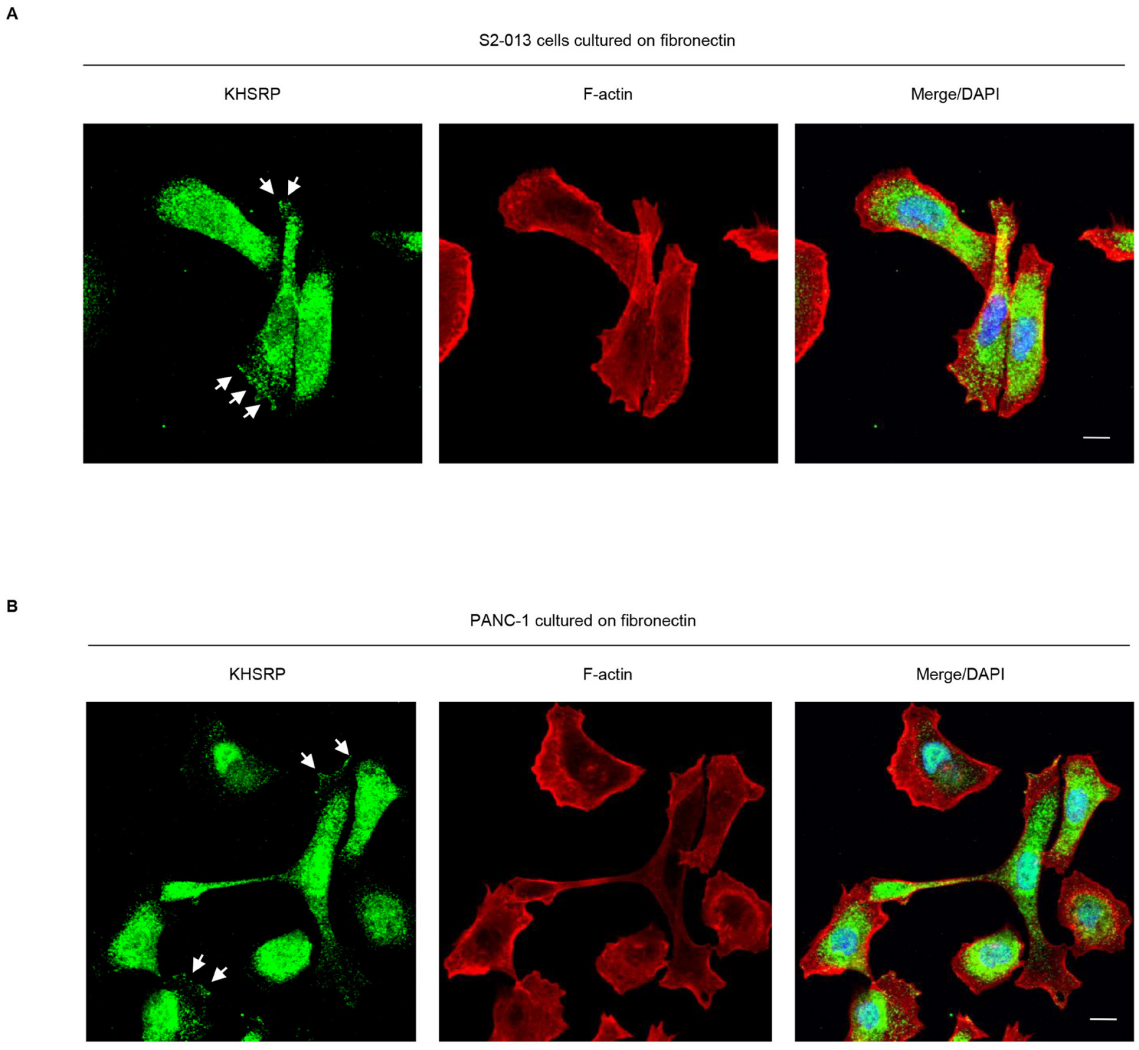
KHSRP distribution in PDAC cells. S2-013 (**A**) and PANC-1 (**B**) cells were incubated on fibronectin and immunocytochemically labeled with anti-KHSRP antibody (green). Actin filaments were labeled by phalloidin (red). Arrows, KHSRP localized in cell protrusions. Blue, DAPI staining. Bars, 10 µm.

We used immunocytochemistry to determine subcellular localization of KHSRP in two types of cultured PDAC cells, moderately differentiated S2-013 PDAC cells and poorly differentiated PANC-1 PDAC cells. KHSRP was localized in the nucleus and cytoplasm, and accumulated in cell protrusions, which had many peripheral actin structures (Figure 1A, 1B).

### Stable knockdown effects of KHSRP on invasiveness and metastasis of PDAC cells

**Figure 2:**
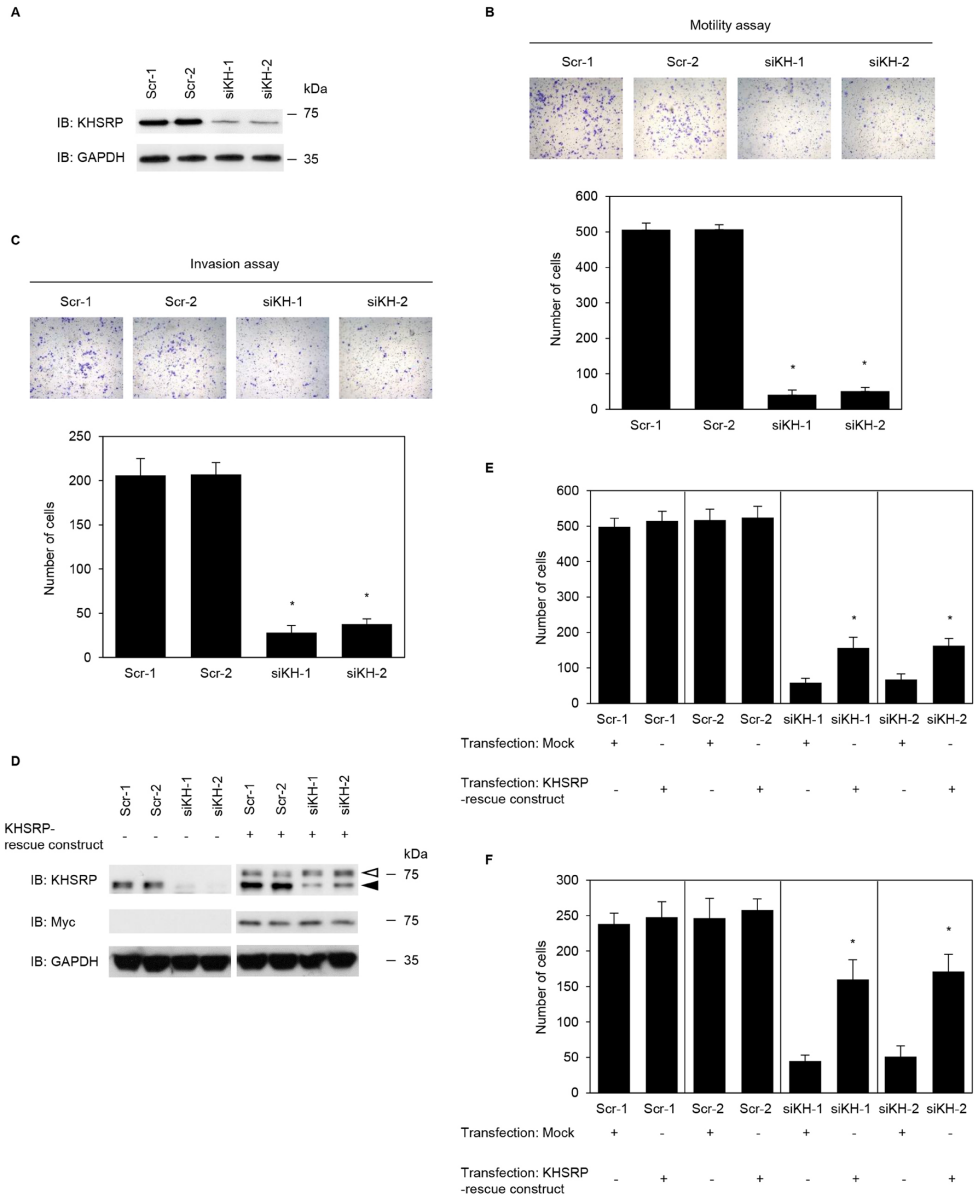
KHSRP promotes cell motility and invasion of PDAC cells. (**A**) Effect of *KHSRP* siRNA in S2-013 cells. Western blots probed with anti-KHSRP antibody show two S2-013 *KHSRP* RNAi clones (siKH-1 and -2) transfected with siRNA targeting *KHSRP* and two scrambled control RNAi clones (Scr-1 and -2). (**B**) Control RNAi or *KHSRP* RNAi S2-013 cells were seeded into two-chamber motility chambers. Migrating cells in four fields per group were counted. Data are derived from three independent experiments and expressed as mean ± SD. ^*^ *p* < 0.05 compared with Scr-1 and Scr-2 (Student’s *t*-test). (**C**) Control RNAi or *KHSRP* RNAi S2-013 cells were seeded into Matrigel invasion chambers. Invading cells in four fields per group were counted. Data are derived from three independent experiments and expressed as mean ± SD. ^*^ *p* < 0.05 compared to Scr-1 or Scr-2 (Student’s *t*-test). (**D**) The mock control vector or myc-tagged *KHSRP*-rescue construct was transiently transfected into control RNAi and *KHSRP* RNAi cells. Western blotting was performed using anti-KHSRP and anti-myc antibodies. Closed arrowhead, endogenous KHSRP; open arrowhead, exogenous KHSRP. Closed arrow head, endogenous KHSRP; open arrow head, exogenous KHSRP. (**E**, **F**) The mock control vector or myc-tagged KHSRP-rescue construct was transiently transfected into control RNAi and *KHSRP* RNAi cells; 48 h later, motility (E) and two-chamber invasion (F) assays were performed. Migrating cells in four fields per group were counted. Data are derived from three independent experiments and expressed as mean ± SD. ^*^ *p* < 0.05 compared with corresponding siKH-1 and siKH-2 transfected mock vector (Student’s *t*-test).

To investigate whether KHSRP affects cell motility and invasion, KHSRP expression in S2-013 cells was stably suppressed by vector-based expression of a *KHSRP* small interfering RNA (siRNA). KHSRP knockdown was confirmed by immunoblotting (Figure 2A). Transwell motility assays showed that motility of S2-013 cells was significantly lower in *KHSRP*-knockdown cells than in scrambled control siRNA-transfected S2-013 cells (Figure 2B). In two-chamber invasion assays, *KHSRP* siRNA-transfected S2-013 cells were significantly less invasive than scrambled control siRNA-transfected S2-013 cells (Figure 2C). Transfection of a KHSRP-rescue construct into *KHSRP* siRNA-transfected S2-013 cells abrogated changes to cell motility and invasiveness caused by transfection of *KHSRP* siRNA (Figure 2D–2F).

An orthotopic murine model of PDAC was used to examine tumor invasiveness and metastasis (Table 1). Incidence of regional invasion of retroperitoneum and peritoneal dissemination by PDAC cells was inhibited in mice injected with *KHSRP* siRNA-transfected S2-013 cells than those injected with scrambled control siRNA-transfected S2-013 cells. Moreover, *KHSRP* siRNA-transfected S2-013 cells did not form hepatic or lung metastases, whereas hepatic and lung metastases were seen in scrambled control siRNA-transfected S2-013 cells. These results indicate that KHSRP specifically promotes invasion and metastasis of PDAC cells.

**Table 1:** Effect of KHSRP-RNAi on invasiveness and metastasis *in vivo*

Cell line	Mice autopsied	Median tumor weight (g) (range)	Peritoneal dissemination	Retroperitoneal invasion	Liver metastasis	Lung metastasis
Scr-1	9	2.7 (1.8–3.5)	7/9	8/9	4/9	3/9
Scr-2	9	2.4 (1.5–3.3)	7/9	9/9	3/9	3/9
siKH-1	10	1.9 (1.2–2.8)	3/10^a^	2/10^a^	0/10^a^	0/10^a^
siKH-2	9	2.1 (1.0–2.9)	2/9^a^	1/9^a^	0/9^a^	0/9^a^

^a^ *p* < 0.05 compared with Scr-1 and Scr-2 (Fisher’s exact test).

### Localization of KHSRP in P-bodies

**Figure 3:**
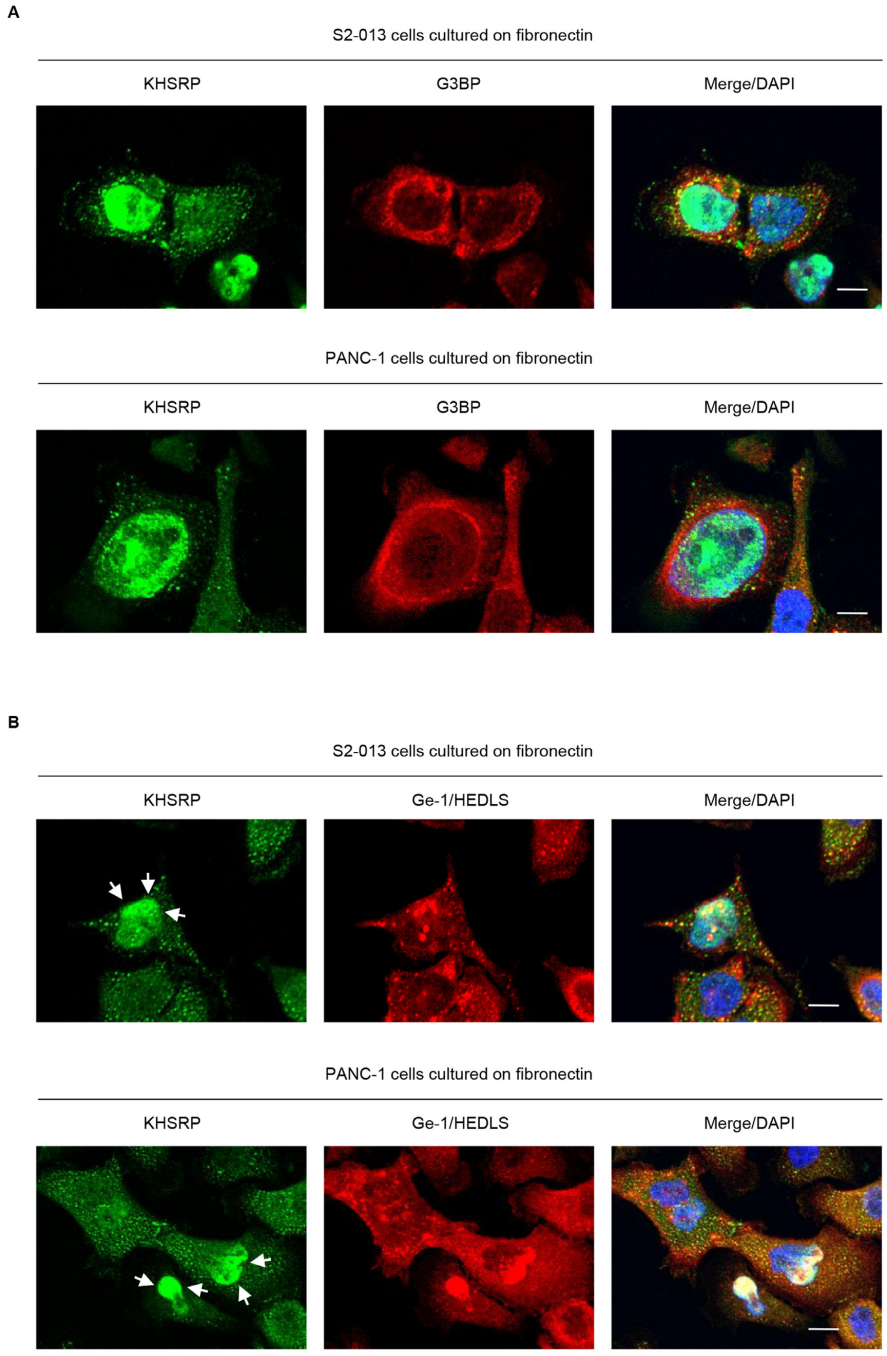
KHSRP localizes in cytoplasmic P-bodies. (**A**, **B**) Confocal immunofluorescence microscopic images. S2-013 and PANC-1 cells were incubated on fibronectin and stained with anti-KHSRP (green) and anti-G3BP (red) (A) or anti-Ge-1/HEDLS (red) (B) antibodies. Arrows, KHSRP localized in the perinuclear region of cytoplasm. Blue, DAPI staining. Bars, 10 µm.

Several types of RNA-binding proteins, 40S subunit ribosomal proteins, and translation initiation factors are present in stress granules [14]. An alternative model for silencing by miRNAs wherein mRNA interactions with RISC sequester targeted mRNAs in processing bodies (P-bodies) was reported [15]. These are cytoplasmic foci that contain non-translated RNAs and exclude the translation machinery [16]. To investigate whether KHSRP-containing granules were stress granules (SGs) or P-bodies, S2-013 and PANC-1 cells cultured on fibronectin were double-labeled with anti-KHSRP, anti-G3BP (a marker for SG) [17], and anti-Ge-1/HEDLS (a marker for P-bodies) [18] antibodies. KHSRP did not colocalize with G3BP in cytoplasmic granules (Figure 3A); however, KHSRP colocalized with P-bodies in a proportion of granules throughout the cytoplasm, as well as in the perinuclear region of cytoplasm (Figure 3B). These data indicate that KHSRP localized in P-bodies may function to regulate levels of certain mRNAs in PDAC cells.

### Identification of target transcripts of KHSRP

**Figure 4:**
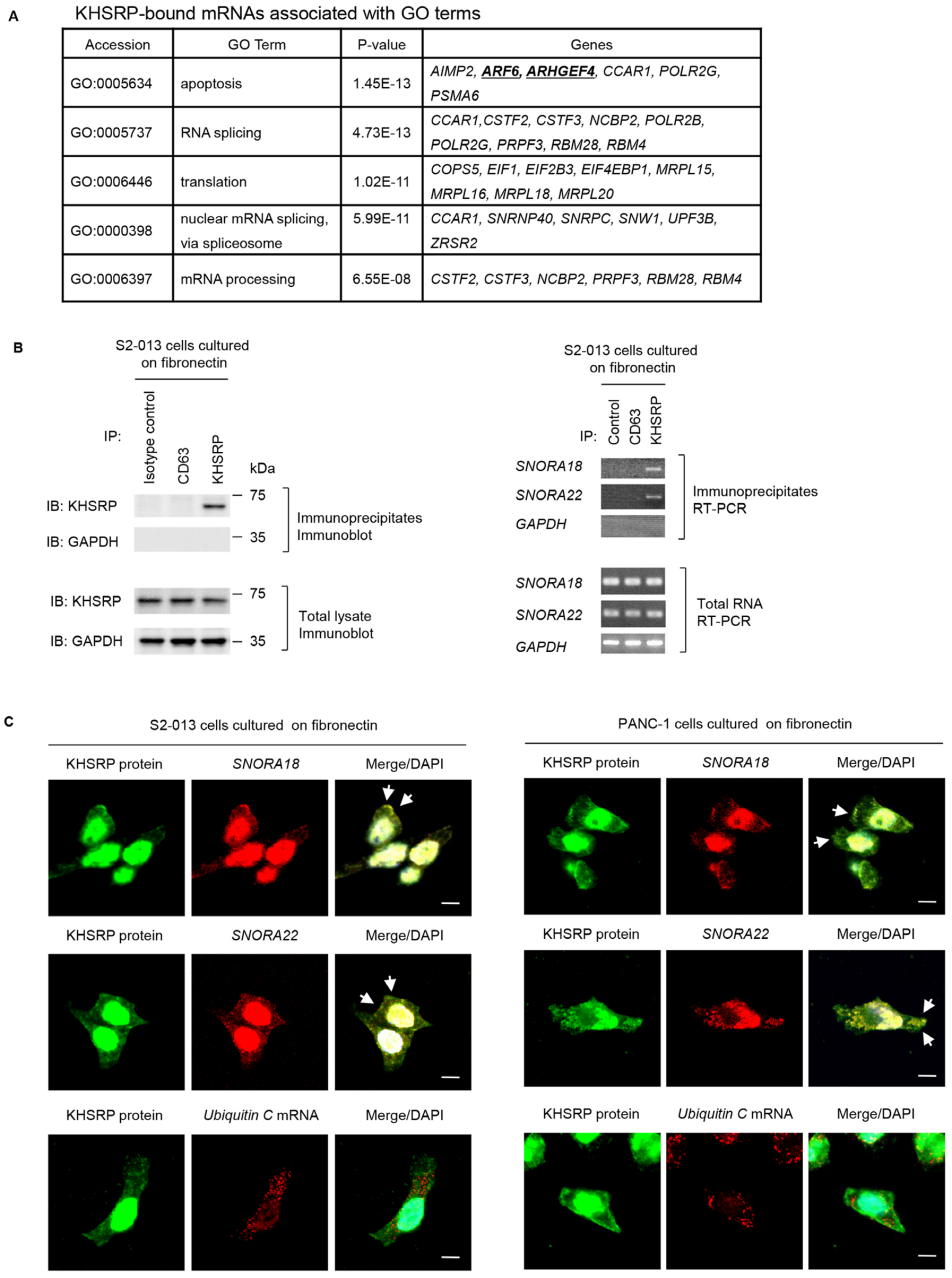
Ultrasequencing analysis of KHSRP-bound transcripts. (**A**) KHSRP-bound transcripts identified in RIP analysis and included in GO terms relevant to cell motility, invasiveness, and protrusions are shown. (**B**) Association between KHSRP and *SNORA18* or *SNORA22* in S2-013 cells cultured on fibronectin was tested via KHSRP-IP or control-IP and subsequent RT-PCR amplification of *SNORA18*, *SNORA22*, and *GAPDH* in the immunoprecipitate (right panels). Proteins in immunoprecipitates were examined on western blots probed with anti-KHSRP and anti-CD63 antibodies (left panels). Rabbit IgG isotype control antibody was used as a negative control for coimmunoprecipitation. (**C**) Colocalization of KHSRP protein (green) and *SNORA18* or *SNORA22* (red) in S2-013 and PANC-1 cells cultured on fibronectin. *Ubiquitin C* mRNA was used as a negative control for colocalization. Arrows, *SNORA18* and *SNORA22* colocalized with KHSRP in cell protrusions. Blue, DAPI staining. Bars, 10 µm.

To identify KHSRP-bound transcripts localized in KHSRP-containing granules, we performed RNA immunoprecipitation (RIP) with anti-KHSRP antibody and cell extracts from S2-013 cells grown on fibronectin, followed by next-generation sequencing to identify any RNAs in resultant immunoprecipitates. Results of RIP assay are presented as log ratios in Supplementary Table 1. We identified 501 RNAs that were significantly enriched in anti-KHSRP immunoprecipitates relative to rabbit IgG isotype control immunoprecipitates (Supplementary Table 1). The complete gene list derived from the 501 RNAs was uploaded onto the Gene Expression Omnibus Database (http://www.ncbi.nlm.nih.gov/geo/) (GEO accession: GSE120853). A considerable number of snoRNAs were listed in the top 10 of KHSRP-bound RNA candidates (Table 2). To gain further insight into biological functionalities of KHSRP-bound RNAs, Gene Ontology (GO) analysis identified GO terms matched to the gene list (*p* < 10^−5^; Supplementary Table 2), and this GO set was significantly enriched with cellular functions relevant to apoptosis, RNA splicing, translation, nuclear mRNA splicing via spliceosome, and mRNA processing. The transcripts that matched any GO term related to these functions are listed in Figure 4A. We previously reported that overexpression of ADP-ribosylation factor 6 (ARF6) and Rho guanine nucleotide exchange factor 4 (ARHGEF4) in PDAC tissue correlates with PDAC-related survival and they promote motility and invasiveness of PDAC cells by increasing cell protrusions [19, 20]. Instead of focusing on the KHSRP-bound mRNAs, the present study showed the functional analysis of KHSRP-bound snoRNAs. Reverse transcription-PCR (RT-PCR) was performed to validate snoRNAs listed in Table 2 were immunoprecipitated with KHSRP (Figure 4B). *SNORA18* and *SNORA22* immunoprecipitated with anti-KHSRP antibody, whereas neither transcript immunoprecipitated with isotype control antibody.

**Table 2:** TOP10 RNAs that co-immunoprecipitate with KHSRP

Gene	Entrez Gene ID	Log ratio RPKM(KHSRP)/RPKM(control IgG)
*SCARNA21* *COX17* *SNORA38* *SNORA25* *SNORA18* *SNORA22* *SNORA14B* *SNORA71B* *SNORA71D* *SNORD97*	677763 10063 677820 684959 677805 677807 677802 26776 677840 692223	3.89 3.60 2.98 2.98 2.95 2.94 2.92 2.91 2.89 2.87

Immunocytochemistry and RNA fluorescence *in situ* hybridization were used together to determine whether *SNORA18* and *SNORA22* colocalize with KHSRP within cytoplasmic granules of S2-013 cells cultured on fibronectin and showed these snoRNAs colocalized with KHSRP (Figure 4C). Control *ubiquitin C* mRNA did not colocalize with KHSRP in S2-013 cells cultured on fibronectin (Figure 4C).

### Roles of KHSRP in formation of cell protrusions

**Figure 5:**
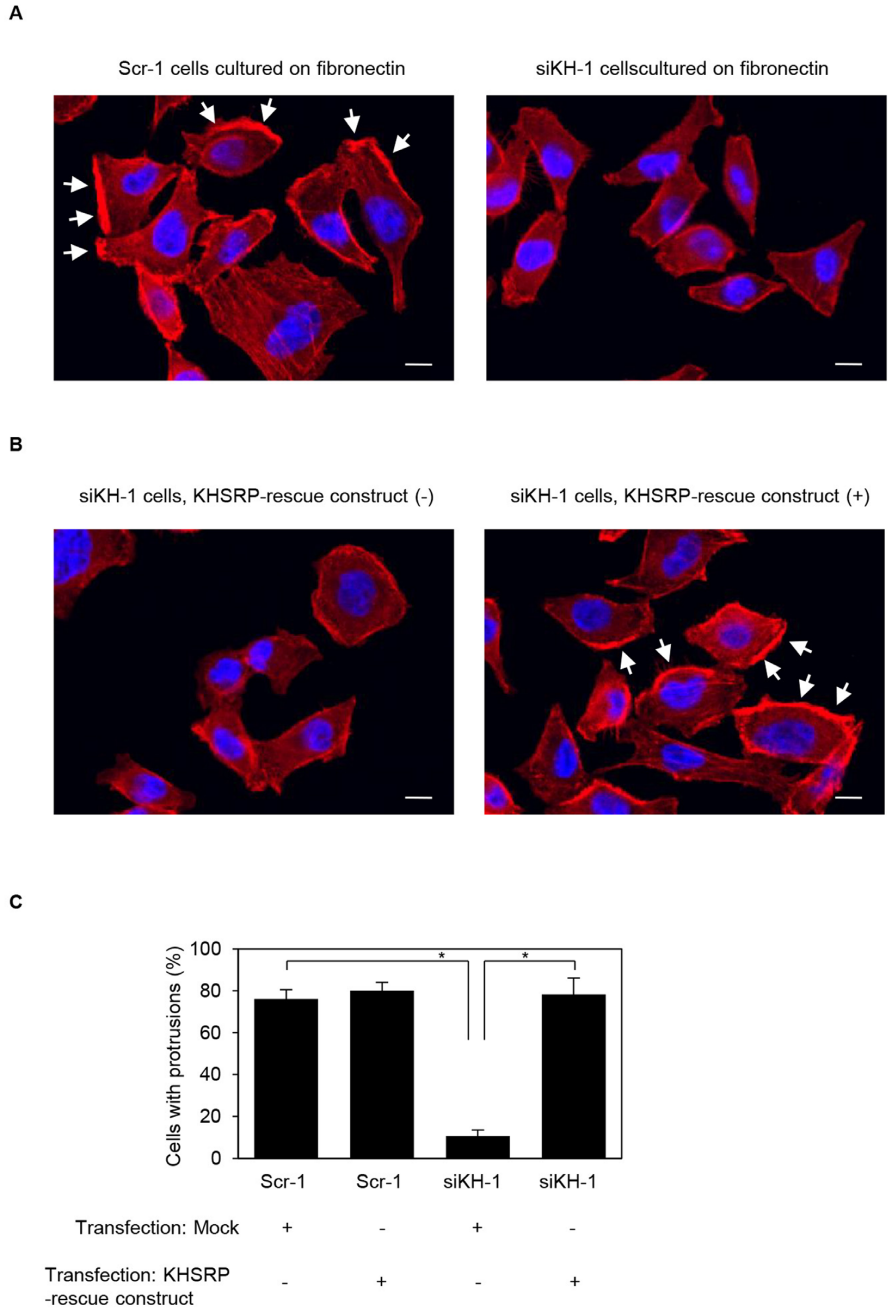
KHSRP associates with forming cell protrusions. (**A**) Confocal immunofluorescence microscopic images show phalloidin-labeled peripheral actin structures (red) and DAPI-labeled nuclei (blue) in scrambled control RNAi (Scr-1) S2-013 cells or *KHSRP* RNAi (siKH-1) S2-013 cells cultured on fibronectin. Arrows, cell protrusions. Bars, 10 µm. (**B**) Confocal immunofluorescence microscopic images. A myc-tagged KHSRP rescue construct was transiently transfected into *KHSRP* RNAi (siKH-1) S2-013 cells and incubated on fibronectin. Red, peripheral actin structures; blue, DAPI-labeled nuclei. Arrows, cell protrusions. Bars, 10 µm. (**C**) Quantification of data shown in Figure 5A and 5B. Values represent the number of cells with fibronectin-mediated cell protrusions in which peripheral actin structures were increased. All cells in four fields per group were scored. Data are derived from three independent experiments and expressed as mean ± SD. ^*^ *p* < 0.05 compared with Scr-1 or siKHSRP-1 transfected mock vector (Student’s *t*-test).

Confocal microscopy showed that peripheral actin structures were less abundant in *KHSRP* siRNA-transfected S2-013 cells than in scrambled control siRNA-transfected S2-013 cells grown on fibronectin (Figure 5A). Conversely, phalloidin-labeled actin structures were more abundant in the cytoplasm of cell bodies of *KHSRP* siRNA-transfected S2-013 cells than that of scrambled control siRNA-transfected S2-013 cells (Figure 5A). Transfection of a KHSRP-rescue construct renewed peripheral actin structures in *KHSRP* siRNA-transfected S2-013 cells (Figure 5B). Cell protrusions were significantly more abundant in *KHSRP* siRNA-transfected S2-013 cells carrying a *KHSRP-*rescue construct than in *KHSRP* siRNA-transfected S2-013 cells not carrying a *KHSRP-*rescue construct (Figure 5C). These results indicate that KHSRP induces rearrangement of peripheral actin to increase formation of cell protrusions.

### Roles of KHSRP-bound snoRNAs in formation of cell protrusions

**Figure 6:**
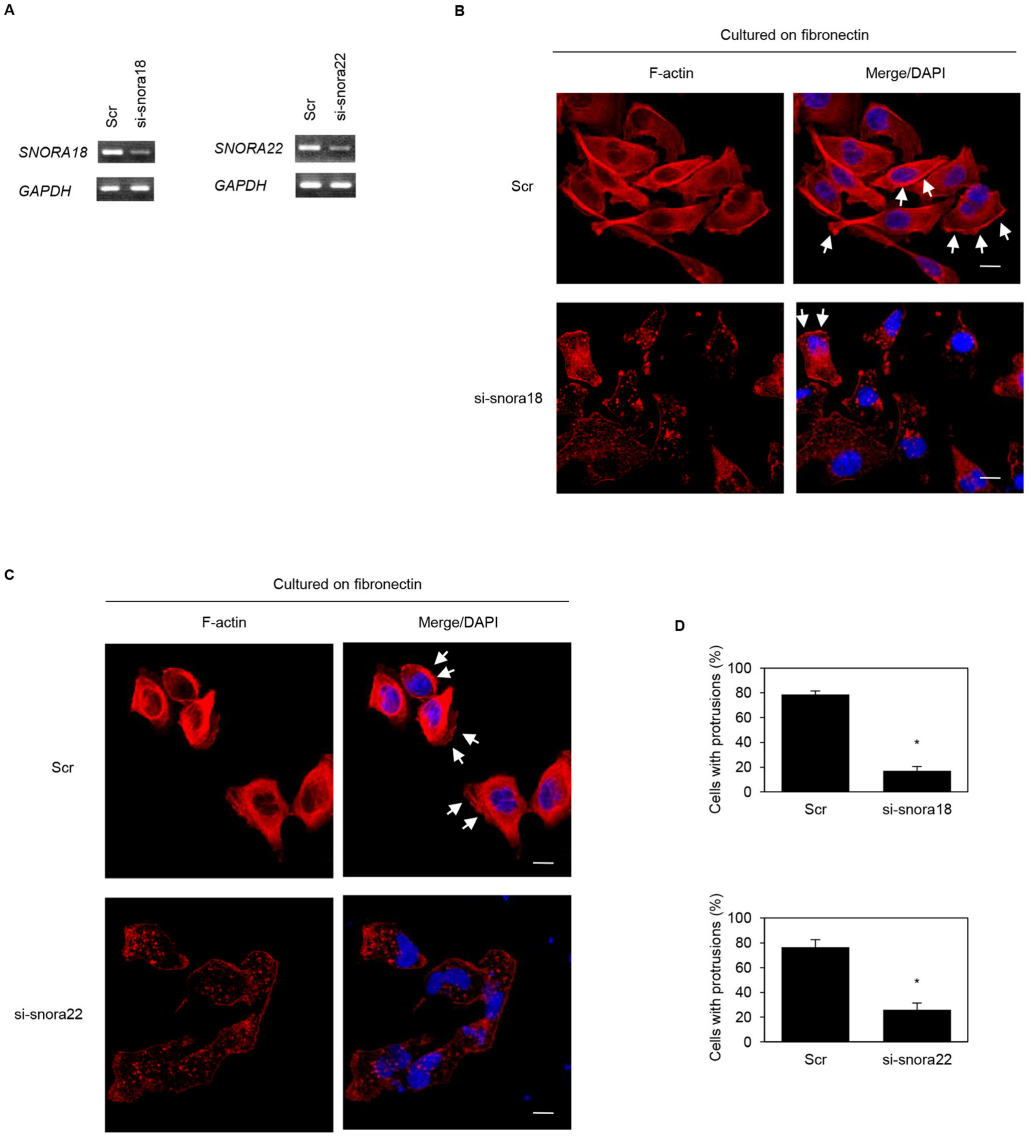
Roles of KHSRP-bound snoRNAs in forming cell protrusions in S2-013 cells. (**A**) siRNA oligonucleotides targeting *SNORA18* (si-snora18) and *SNORA22* (si-snora22) or negative control scrambled siRNAs (Scr) were transiently transfected into S2-013 cells. Semi-quantitative RT-PCR was performed using specific primer sets. (**B**, **C**) si-snora18, si-snora22, or Scr was transiently transfected into S2-013. After 48 h, cells were incubated on fibronectin. Actin filaments were labeled by phalloidin (red) and DAPI (blue). si-snora18-transfected S2-013 and si-snora22-transfected S2-013 cells are shown in (B) and (C), respectively. Arrows, cell protrusions. Bars, 10 µm. (**D**) Quantification of data shown in Figure 6B and 6C. Values represent the number of cells with fibronectin-mediated cell protrusions in which peripheral actin structures were increased. All cells in four fields per group were scored. Data are derived from three independent experiments and expressed as mean ± SD. ^*^ *p* < 0.05 compared with scrambled control siRNA-transfected S2-013 cells (Student’s *t*-test).

**Figure 7:**
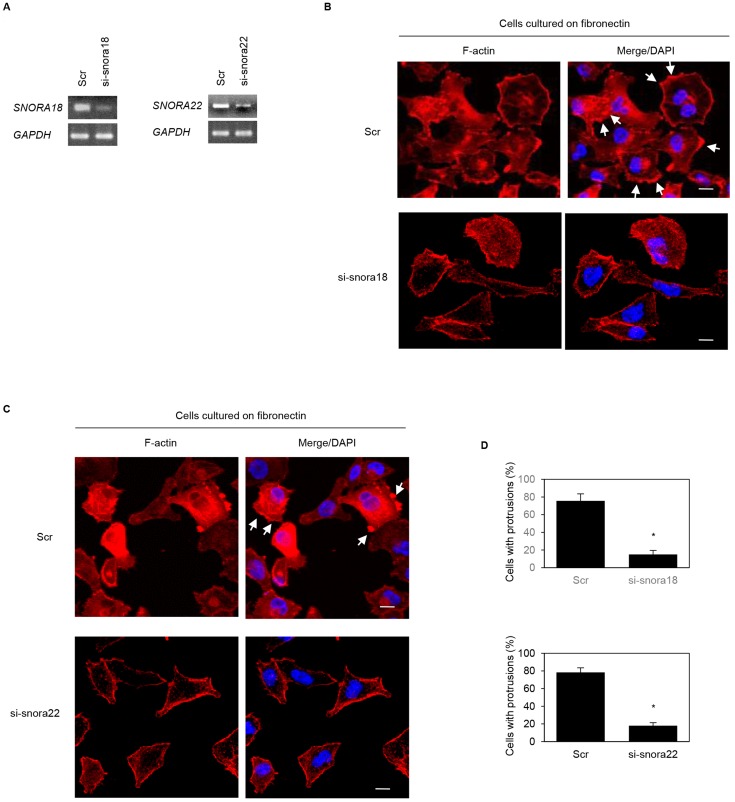
Roles of KHSRP-bound snoRNAs in forming cell protrusions in PANC-1 cells. (**A**) siRNA oligonucleotides targeting *SNORA18* (si-snora18) and *SNORA22* (si-snora22) or negative control scrambled siRNAs (Scr) were transiently transfected into PANC-1 cells. Semi-quantitative RT-PCR was performed using specific primer sets. (**B**, **C**) si-snora18, si-snora22, or Scr was transiently transfected into PANC-1. After 48 h, cells were incubated on fibronectin. Actin filaments were labeled by phalloidin (red) and DAPI (blue). si-snora18-transfected PANC-1 and si-snora22-transfected PANC-1 cells are shown in (B) and (C), respectively. Arrows, cell protrusions. Bars, 10 µm. (**D**) Quantification of data shown in Figure 7B and 7C. Values represent the number of cells with fibronectin-mediated cell protrusions in which peripheral actin structures were increased. All cells in four fields per group were scored. Data are derived from three independent experiments and expressed as mean ± SD. ^*^ *p* < 0.05 compared with scrambled control siRNA-transfected PANC-1 cells (Student’s *t*-test).

We previously reported that ARF6 and ARHGEF4 proteins, whose mRNAs are bound to an RNA-binding protein insulin-like growth factor 2 mRNA binding protein 3 (IGF2BP3), accumulate in cell protrusions and thereby contribute to cell protrusion formation in PDAC cells [19, 20]. To determine whether KHSRP-bound snoRNAs, *SNORA18* and *SNORA22*, participate in induction of membrane protrusions, we analyzed peripheral actin structures in membrane ruffles of S2-013 and PANC-1 cells transiently transfected with scrambled control siRNA, *SNORA18* siRNA, and *SNORA22* siRNA. Based on semi-quantitative RT-PCR data, 72 h after transfection, *SNORA18* and *SNORA22* expression was markedly higher in scrambled control siRNA-transfected S2-013 and PANC-1 cells cultured on fibronectin than in *SNORA18* siRNA-transfected or *SNORA22* siRNA-transfected S2-013 and PANC-1 cells cultured on fibronectin (Figures 6A, 7A). Confocal microscopy revealed that *SNORA18*- or *SNORA22*-knockdown in S2-013 and PANC-1 cells cultured on fibronectin decreased peripheral actin structures (Figures 6B, 7B and Figures 6C, 7C). Furthermore, *SNORA18*- and *SNORA22*-knockdown in S2-013 and PANC-1 cells cultured on fibronectin significantly inhibited formation of cell protrusions (Figures 6D, 7D). These results indicate that *SNORA18* and *SNORA22* play roles in formation of cell protrusions.

### Roles of KHSRP-bound snoRNAs in cell motility and invasiveness of PDAC cells

**Figure 8:**
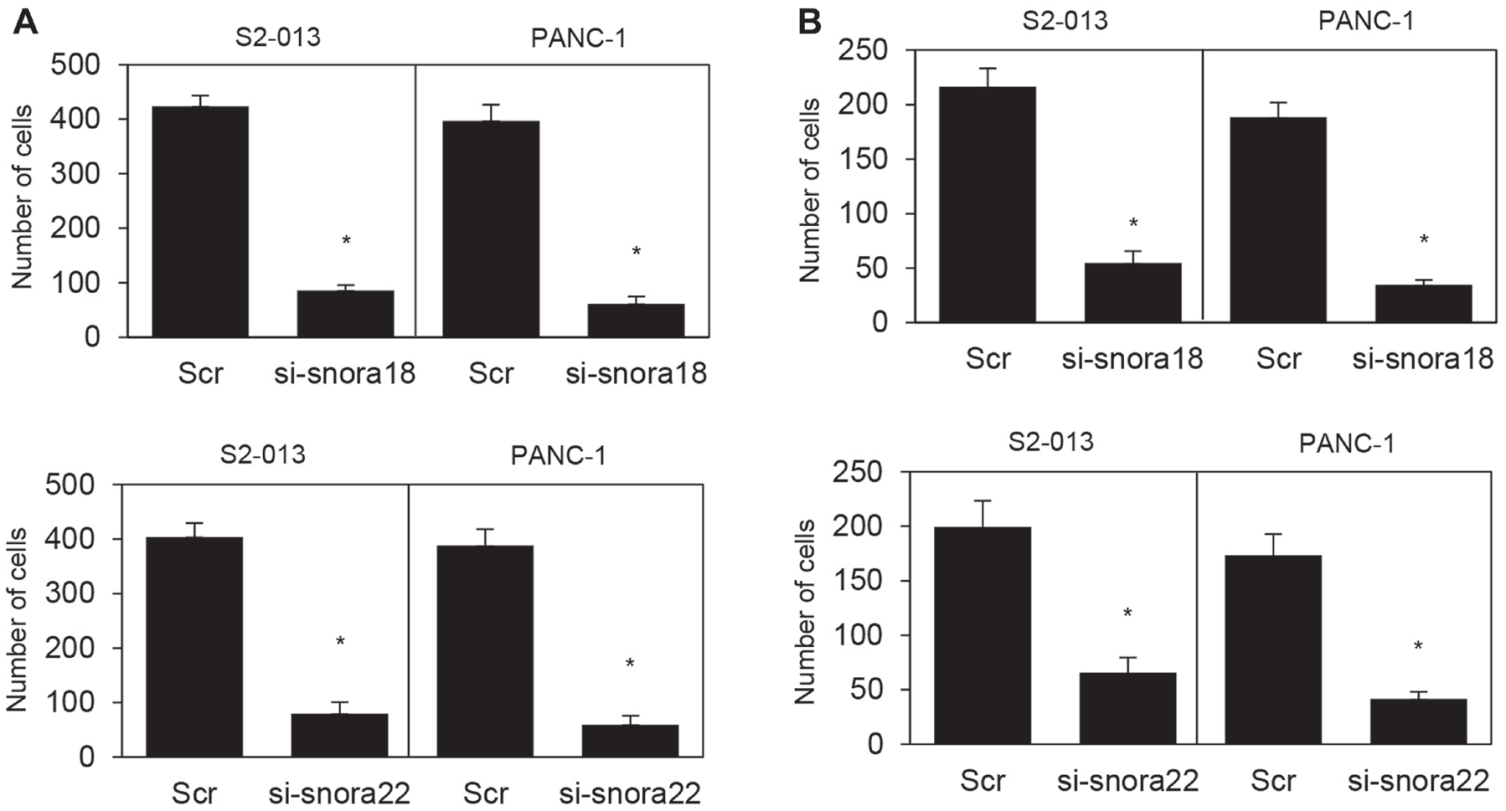
Roles of KHSRP-bound snoRNAs in cell motility and invasion. (**A**, **B**) si-snora18, si-snora22, or Scr was transiently transfected into S2-013 and PANC-1 cells. Motility (A) and two-chamber invasion (B) assays were performed. Migrating cells in four fields per group were scored. Data are derived from three independent experiments and expressed as mean ± SD. ^*^ *p* < 0.05 compared with scrambled control siRNA-transfected S2-013 cells (Student’s *t*-test).

In transwell motility assays, motility of S2-013 cells and PANC-1 cells was significantly lower in *SNORA18*- or *SNORA22*-knockdown cells than in scrambled control cells (Figure 8A). In two-chamber invasion assays, invasiveness of S2-013 and PANC-1 cells was significantly lower in *SNORA18*- or *SNORA22*-knockdown cells than in scrambled control cells (Figure 8B). These results indicate that *SNORA18* and *SNORA22* associate with promotion of PDAC cell motility and invasiveness.

### Effects of knockdown of SNORA18 and SNORA22 on invasiveness and metastasis in the orthotopic murine model of PDAC

**Figure 9:**
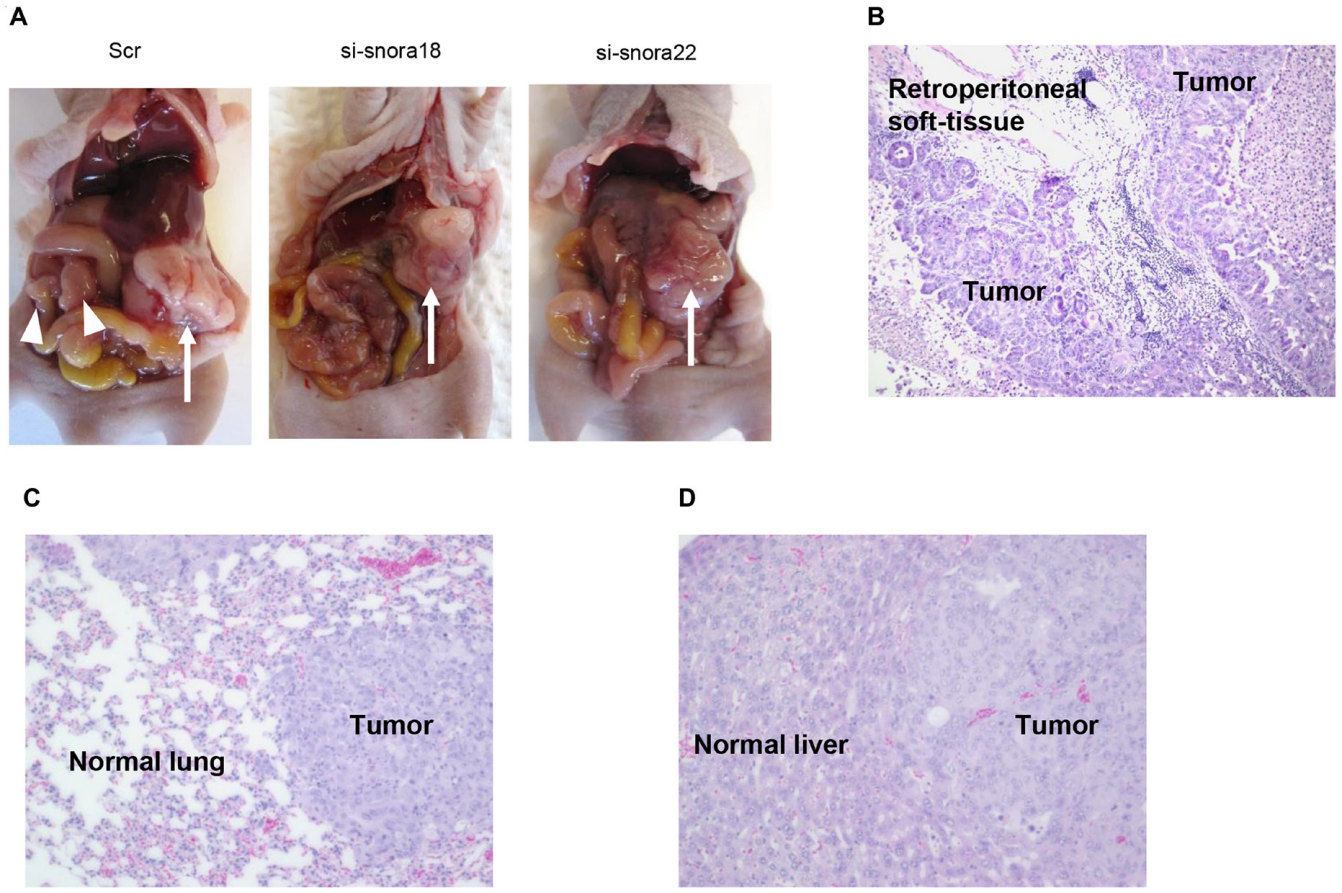
Knockdown effect of siRNA-FA-PEG-COL nanoparticles targeting KHSRP-bound snoRNAs on cell motility and invasion in the orthotopic murine model of PDAC. (**A**) Development of carcinomatosis in S2-013 tumor-bearing mice treated with scrambled control siRNA-FA-PEG-COL nanoparticles (Scr) and target siRNA-FA-PEG-COL nanoparticles against *SNORA18* (si-snora18) and *SNORA22* (si-snora22). Arrow, primary tumor; arrowheads, dissemination nodules in the abdominal cavity. (**B**–**D**) Hematoxylin and eosin staining of representative sections of S2-013-derived PDAC tumor tissues in mice treated with scrambled control siRNA-FA-PEG-COL nanoparticles showing areas of regional invasion of the retroperitoneum (B) and distant metastases to the lung (C) and liver (D). Original magnification: 200 ×.

*In vivo* imaging studies showed that scrambled control siRNA-folic acid (FA)-modified polyethylene glycol (PEG)-chitosan oligosaccharide lactate (COL) nanoparticles, which were intravenously injected into the orthotopic murine model of PDAC [19, 21], were taken up into PDAC cells in S2-013-derived PDAC tumor of the mouse pancreas [22]. To study the effects of *SNORA18* and *SNORA22* on invasiveness and metastasis *in vivo*, siRNA-FA-PEG-COL nanoparticles against *SNORA18* and *SNORA22* were intravenously injected into the orthotopic murine model of PDAC. On day 4 postimplantation of S2-013 cells into the pancreas of each mouse, nanoparticles against *SNORA18* and *SNORA22* or control solutions were intravenously injected into mice in each group. All mice received a total of five intravenous injections once a week. Six weeks after implantation, mice were euthanized, and hematoxylin and eosin staining was performed on sections of PDAC tissues, lung, and liver to determine the presence of retroperitoneum invasion, peritoneal dissemination, and metastasis to the liver and lung. Extensive peritoneal carcinomatosis was seen in 70% of mice in control groups (Figure 9A), and hematoxylin and eosin staining of S2-013-derived PDAC tumors showed adenocarcinoma with regional invasion of the retroperitoneum (Figure 9B). Hematoxylin and eosin staining of liver and lung metastases of control mice is shown in Figure 9C and 9D, respectively. siRNA-FA-PEG-COL nanoparticles against *SNORA18* and *SNORA22* significantly inhibited retroperitoneal invasion and lung metastasis compared with control groups (Table 3). Notably, siRNA-FA-PEG-COL nanoparticles against *SNORA18* strongly inhibited retroperitoneal invasion.

**Table 3:** Effect of the target siRNA nanoparticles on invasiveness and metastasis *in vivo*

	Mice (*n*)	Peritoneal dissemination	Retroperitoneal invasion	Liver metastasis	Lung metastasis
PBS	11	8/11	9/11	6/11	6/11
Scrambled control siRNA-COL	10	6/10	9/10	4/10	8/10
Scrambled control siRNA-FA-PEG-COL nanoparticles	9	6/9	7/9	3/9	6/9
*SNORA18* siRNA-FA-PEG-COL nanoparticles	10	6/10	0/10^a^	5/10	2/10^a^
*SNORA22* siRNA-FA-PEG-COL nanoparticles	8	3/8	2/8^a^	2/8	3/8^a^

^a^ *p* < 0.05 compared to controls (PBS, FA-PEG-COL nanoparticles and scrambled control siRNA)

## DISCUSSION

Here, we found novel functions for KHSRP in PDAC cells. First, KHSRP prompts invasiveness and metastasis of PDAC cells. Second, KHSRP binds a specific set of snoRNAs in cytoplasmic P-bodies. Finally, siRNA-FA-PEG-COL nanoparticles targeting these KHSRP-bound snoRNAs inhibit PDAC invasion and metastasis.

SGs containing another RNA-binding protein, IGF2BP3, also accumulate in cell protrusions, which promote invasion and metastasis of PDAC cells via regulation of localized translation of IGF2BP3-bound mRNAs in cell protrusions [19]. Here, we found that KHSRP accumulated in cytoplasmic P-bodies but not in SGs of PDAC cells, indicating that KHSRP-bound snoRNAs could accumulate in P-bodies. siRNA and miRNA are involved in mRNA degradation or translational arrest in cytoplasmic P-bodies [23]. snoRNA U3-small nucleolar ribonucleoprotein complex shuttles between the nucleolus and cytoplasm [24]. The presence of box C/D snoRNAs in the cytoplasm raises the possibility that snoRNAs might target cytoplasmic RNAs [25]. These findings suggest that cytoplasmic snoRNAs have different roles in addition to ribosome biogenesis in the nucleolus. Thus, KHSRP-bound snoRNAs may be present in P-bodies as distinct complexes in PDAC cells. Future studies are needed to elucidate the mechanism by which KHSRP recognizes its different snoRNA targets in P-bodies of PDAC cells.

KHSRP is a novel Dishevelled-interacting protein, which is associated with Wnt/β-catenin signaling pathway element dishevelled segment polarity protein 3 (DVL3) and acts as a negative regulator of Wnt/β-catenin signaling through post-transcriptional regulation of *β-catenin* mRNA [26]. Deregulation of Wnt/β-catenin signaling is implicated in PDAC and promotes cell invasion and metastasis of PDAC [27]. Suppression of DVL3 enhances basal RAS-MEK-ERK activation and activates IGF signal transduction from the IGF1 receptor to RAS [28]. AKT phosphorylates KHSRP, and phosphorylated KHSRP binds to a molecular chaperone 14-3-3, which in turn decreases KHSRP’s ability to promote *β-catenin* mRNA decay [5]. We recently reported that coiled-coil domain containing 88A (CCDC88A) induces formation of membrane protrusions and increases migration and invasiveness of PDAC cells [29]. CCDC88A phosphorylation by AKT occurs at the leading edge, which is required for cancer cell invasion and metastasis [30]. These reports suggest that Wnt/β-catenin and AKT contribute to invasiveness and metastasis of PDAC cells, and both signaling pathways regulate KHSRP activity that promotes *β-catenin* mRNA decay. Therefore, post-transcriptional regulation of the KHSRP’s target mRNAs may play important roles in modulating the invasiveness and metastasis of PDAC cells. It is interesting to consider the possibility that KHSRP-bound snoRNAs post-transcriptionally regulate levels of target mRNAs, and in turn they promote invasiveness and metastasis of PDAC cells.

KHSRP interacts with a broad range of AU-rich sequences and recognizes a subset of AREs, recruiting the exosome and de-adenylation factors to mRNA targets for cell proliferation [2, 31]. The HuR-KHSRP complex downregulates *nucleophosmin* mRNA and induces commitment of muscle cells to myogenesis [32]. p38-mediated phosphorylation of KHSRP post-transcriptionally downregulates *utrophin A* mRNA in skeletal muscle and may also enhance muscle regeneration [33]. The present study showed that KHSRP-bound snoRNAs associated with formation of cell protrusions and promotion of PDAC cell motility and invasion. KHSRP destabilizes mRNAs such as *myogenin* through direct binding to a long noncoding RNA H19 in the cytoplasm [34]. We hypothesize that KHSRP-snoRNA complexes can recognize a specific set of mRNAs to promote PDAC cell invasion and metastasis. KHSRP binds to the terminal loop of target miRNA precursors and induces their maturation [35]. This mechanism is required for post-translational regulation of target mRNAs of KHSRP-miRNA complexes that affect specific biological processes, including proliferation, apoptosis, and differentiation [35]. snoRNAs are linked to lipotoxicity [36]. Protein kinase RNA-activated (PKR) binds to snoRNAs during cellular metabolic stress, and snoRNAs activate PKR during metabolic stress [37]. Future studies are needed to delineate the mechanism that links KHSRP to the machinery regulating maturation of snoRNAs and determine whether KHSRP-snoRNA complexes contribute to target mRNA decay in P-bodies of PDAC cells.

The data presented herein showed that siRNA-FA-PEG-COL nanoparticles against *SNORA18* and *SNORA22* significantly inhibited regional invasion of the retroperitoneum and peritoneal dissemination. Moreover, siRNA-FA-PEG-COL nanoparticles against *SNORA18* strongly inhibited retroperitoneal invasion. Thus, these siRNA nanoparticles could be useful for discovering better approaches for patients with PDAC. The functional importance of the association of KHSRP with snoRNAs mediated regulation of the invasiveness and metastasis in PDAC cells suggests that inhibition of KHSRP expression, binding of KHSRP with snoRNAs, or expression of KHSRP-bound snoRNAs such as *SNORA18* and *SNORA22* may be effective for PDAC targeted molecular therapy.

## MATERIALS AND METHODS

### Antibodies

Anti-KHSRP antibody (ab140648) was purchased from Abcam (Cambridge, MA, USA). Anti-G3BP monoclonal antibody (611126) was purchased from BD Transduction Laboratory (Palo Alto, CA, USA). Anti-c-myc (sc-40) and anti-Ge-1/HEDLS (sc-8418) antibodies were purchased from Santa Cruz Biotechnology (Santa Cruz, CA, USA).

### Cell culture and reagents

The human PDAC cell line S2-013, a subline of SUIT-2, was donated by Dr. Michael Hollingsworth at the University of Nebraska (Omaha, NE, USA). The human PDAC cell line PANC-1 was obtained from the American Type Culture Collection (Manassas, VA, USA). All cells were maintained in Dulbecco’s modified Eagle’s medium (Gibco; Thermo Fisher Scientific, Inc., Waltham, MA, USA) containing 10% fetal calf serum (Gibco) at 37°C.

### Generation of a S2-013 cell line that stably expresses KHSRP siRNA

S2-013 cells, in which KHSRP expression was stably suppressed by vector-based expression of a *KHSRP* siRNA (TR311984; OriGene Technologies, Rockville, MD, USA), were generated as detailed previously [19]. Cells were used only when suppression of KHSRP expression had been validated via western blot analysis.

### KHSRP-rescue construct

RT-PCR of total RNA extracted from S2-013 cells was used to amplify the entire coding sequence of *KHSRP* cDNA. The resultant PCR product was cloned into pCMV6-Entry vector (OriGene Technologies Inc.) bearing a C-terminal myc-DDK tag. Transient transfection of the resultant KHSRP-rescue construct was carried out using X-tremeGENE HP DNA Transfection Reagent (Roche Applied Science, Penzberg, Germany) at room temperature. Transfected cells were assayed typically 2 days following transfection.

### Immunoblotting analysis of cell lysates

Total cell lysates were extracted using lysis buffer [Tris-HCl (pH 7.4), sodium dodecyl sulfate (SDS), mercaptoethanol, and glycerol]. Protein concentrations were determined using Takara Bradford Protein Assay kit (T9310A; Takara Bio, Shiga, Japan), and an equal amount of protein (10 µg) was separated on a 4–20% gradient SDS-PAGE (TEFCO, Tokyo, Japan). Proteins were transferred using a Trans-Blot Turbo RTA Mini LF PVDF Transfer kit (170-4274; Bio-Rad Laboratories, Hercules, CA, USA) to a Trans-Blot Turbo Mini-size LF polyvinylidene difluoride membrane (Bio-Rad Laboratories) and blocked with 5% non-fat dry milk in Tris-buffered saline [10 mM Tris-HCl (pH 7.4), 150 mM NaCl, 0.1% Tween-20] for 1 h at room temperature. Membranes were then incubated with anti-KHSRP and anti-myc primary antibodies at dilutions of 1:1,000 in 5% non-fat dry milk in Tris-buffered saline overnight at 4°C. Following incubation with appropriate secondary antibodies conjugated with horseradish peroxidase (sc-2004, sc-2005; Santa Cruz Biotechnology) at dilutions of 1:2,000 for 1 h at room temperature, immunoreactive bands were visualized using the ECL Plus kit (GE Healthcare, Chicago, IL, USA) according to the manufacturer’s instructions.

### Immunoprecipitation

Immunoprecipitation was performed as described previously [38].

### RNA immunoprecipitation, next-generation sequencing, and bioinformatics analysis

RIP-sequencing and bioinformatics analysis were performed as described previously [19]. Purified RNAs obtained via RIP were subjected to reverse transcription with StrataScript reverse transcriptase (Agilent, La Jolla, CA, USA) and random primers. We prepared appropriate dilutions of each single-stranded cDNA for subsequent PCR amplification; *GAPDH* was used as an internal quantitative control. We regarded transcripts with a log_2_ ratio (RPKM from KHSRP sample/RPKM from isotype control sample) > 1.0 as transcripts that potentially bound KHSRP (2,826 genes). GO analyses were performed for genes represented by KHSRP-bound transcripts. Statistically significant biological process terms were obtained using Pathway Studio (Ariadne Genomics, Inc., Rockville, MD, USA) [39] by World Fusion Co., Ltd (Tokyo, Japan).

### Immunofluorescence with RNA fluorescence *in situ* hybridization

The QuantiGene ViewRNA plate-based assay kit (Panomics, Santa Clara, CA, USA) was used according to the manufacturer’s recommendations with some modifications [40] to perform fluorescence *in situ* hybridization to target RNAs. Fibronectin-stimulated S2-013 cells were fixed in 8% formaldehyde, dehydrated in an ethanol series (50%, 70%, 100%), and held at 4°C overnight. Cells were then rehydrated, permeabilized, and hybridized as recommended. snoRNA targets were *SNORA18* and *SNORA22* (Panomics), and reference RNA was *ubiquitin C* (Panomics). After *in situ* hybridization, sections were washed in PBS, blocked for 1 h with blocking buffer (4% goat serum in PBS), and incubated for 3 h at room temperature with anti-KHSRP antibody in blocking buffer. Secondary antibodies in blocking buffer were applied to the samples for 30 min at room temperature, nuclei were stained for 3 min with DAPI, and samples were mounted in Aqua-Poly/Mount (Polysciences, Warrington, PA, USA). Confocal fluorescence images were captured with a Zeiss LSM 510 META microscope (Gottingen, Germany).

### siRNA treatment

Mixtures of four different siRNA oligonucleotides that targeted *SNORA18* or *SNORA22* were purchased from Qiagen (FlexiTube GeneSolution: 3046499 and 2650095, respectively; Valencia, CA), and mixtures of four different scrambled negative control siRNA oligonucleotides were purchased from Santa Cruz Biotechnology (37007). To examine the effect of a siRNA on *SNORA18* or *SNORA22* expression, S2-013 cells were plated in six-well plates. After 20 h, cells were transfected with 80 pmol of siRNA in siRNA transfection reagent (Qiagen) following the manufacturer’s instructions. After 48-h incubation, cells were used for immunocytochemistry, transwell motility assays, or Matrigel invasion assays.

### Semi-quantitative RT-PCR

Total RNAs were extracted from each cell line using TRIzol reagent (Invitrogen Life Technologies, Carlsbad, CA, USA) according to the manufacturer’s recommendations, treated with DNase I (Roche Applied Science), and reversely transcribed into single-stranded cDNAs using StrataScript reverse transcriptase (Agilent) and random primers. We prepared appropriate dilutions of each single-stranded cDNA for subsequent PCR amplification by monitoring *GAPDH* as a quantitative control. All reactions involved initial denaturation at 94°C for 2 min followed by 21 cycles (for *GAPDH*) or 28 cycles (for targets) at 94°C for 30 s, 58°C for 30 s, and 72°C for 1 min, on a GeneAmp PCR system 9700 (PE Applied Biosystems, Foster, CA, USA).

### Confocal immunofluorescence microscopy

Immunocytochemistry was carried out as described previously [21, 22].

### Transwell motility assay

Transwell motility assay was carried out, as detailed previously [19].

### Matrigel invasion assay

Matrigel invasion assay was carried out as described previously [19].

### Synthesis of the FA-PEG-COL conjugate

The FA-PEG-COL conjugate was synthesized as described previously [41].

### Fabrication of nanoparticles

Fabrication of nanoparticles was carried out as described previously [41].

### Preparation of siRNA-loaded COL and FA-PEG-COL nanoparticles

siRNAs against *SNORA18* (5′-UUUACUUUACUCACAGGACUA-3′), *SNORA22* (5′-CUUGGCUUUGACCCUGUGCUA-3′), and a scrambled control siRNA (5′-UUCUCCGAACGUGUCACGUAU-3′) were synthesized by GeneDesign (Osaka, Japan). siRNA-loaded COL and FA-PEG-COL nanoparticles were synthesized as described previously [22].

### Mice and orthotopic implantation of tumor cells

Orthotopic tumor induction was performed as described previously [19, 21]. All experiments were performed in accordance with the Institutional Animal Care and Use Committee guidelines of Kochi University. Six-week-old immunocompetent female BALB/cSlc-*nu/nu* mice were obtained from Japan SLC, Inc. (Shizuoka, Japan). Cells (8.0 × 10^5^) in a total volume of 10 µL consisting of equal volumes of PBS were surgically and slowly injected into the head of the pancreas under avertin (0.375 g/kg intraperitoneally) anesthesia. Each mouse was euthanized 42 days after the respective implantation. The presence of tumor invasion into the retroperitoneum and of metastatic lesions in the lung and liver was analyzed by hematoxylin and eosin staining. Pancreatic tumors were excised, examined, and weighed.

In experiments using siRNA-loaded COL and FA-PEG-COL nanoparticles, mice were treated with siRNA-loaded FA-PEG-COL nanoparticles once per week for 6 weeks as detailed previously [22].

### Statistical analysis

The significance of differences between groups was determined using the two-tailed Student’s *t*-test or Fisher’s exact test, as appropriate. Statistical analyses were conducted using GraphPad Prism software (version 6.0, GraphPad Software, Inc., La Jolla, CA). For all analyses, *p* < 0.05 was considered statistically significant.

## SUPPLEMENTARY MATERIALS





## Abbreviations

PDAC: pancreatic ductal adenocarcinoma
KHSRP: KH-type splicing regulatory protein
ARE: AU-rich element
P-body: processing body
miRNA: microRNA
snoRNA: small nucleolar RNA
RT-PCR: reverse transcription-PCR
FA-PEG-COL: folic acid-modified polyethylene glycol-chitosan oligosaccharide lactate

## References

[1] Kiebler MA , Des Groseillers L . Molecular insights into mRNA transport and local translation in the mammalian nervous system. Neuron. 2000; 25:19–28. 10.1016/S0896-6273(00)80868-5. 10707969

[2] Chen CY , Gherzi R , Ong SE , Chan EL , Raijmakers R , Pruijn GJ , Stoecklin G , Moroni C , Mann M , Karin M . AU binding proteins recruit the exosome to degrade ARE-containing mRNAs. Cell. 2001; 107:451–464. 10.1016/S0092-8674(01)00578-5. 11719186

[3] Briata P , Chen CY , Giovarelli M , Pasero M , Trabucchi M , Ramos A , Gherzi R . KSRP, many functions for a single protein. Front Biosci. 2011; 16:1787–1796. 10.2741/3821. 21196264

[4] Briata P , Forcales SV , Ponassi M , Corte G , Chen CY , Karin M , Puri PL , Gherzi R . p38-dependent phosphorylation of the mRNA decay-promoting factor KSRP controls the stability of select myogenic transcripts. Mol Cell. 2005; 20:891–903. 10.1016/j.molcel.2005.10.021. 16364914

[5] Gherzi R , Trabucchi M , Ponassi M , Ruggiero T , Corte G , Moroni C , Chen CY , Khabar KS , Andersen JS , Briata P . The RNA-binding protein KSRP promotes decay of beta-catenin mRNA and is inactivated by PI3K-AKT signaling. PLoS Biol. 2006; 5:e5. 10.1371/journal.pbio.0050005. 17177604 PMC1702562

[6] Trabucchi M , Briata P , Garcia-Mayoral M , Haase AD , Filipowicz W , Ramos A , Gherzi R , Rosenfeld MG . The RNA-binding protein KSRP promotes the biogenesis of a subset of microRNAs. Nature. 2009; 459:1010–1014. 10.1038/nature08025. 19458619 PMC2768332

[7] Min H , Turck CW , Nikolic JM , Black DL . A new regulatory protein, KSRP, mediates exon inclusion through an intronic splicing enhancer. Genes Dev. 1997; 11:1023–1036. 10.1101/gad.11.8.1023. 9136930

[8] Huang S . Review: perinucleolar structures. J Struct Biol. 2000; 129:233–240. 10.1006/jsbi.2000.4247. 10806073

[9] Hoek KS , Kidd GJ , Carson JH , Smith R . hnRNP A2 selectively binds the cytoplasmic transport sequence of myelin basic protein mRNA. Biochemistry. 1998; 37:7021–7029. 10.1021/bi9800247. 9578590

[10] Kiebler MA , Hemraj I , Verkade P , Köhrmann M , Fortes P , Marión RM , Ortín J , Dotti CG . The mammalian staufen protein localizes to the somatodendritic domain of cultured hippocampal neurons: implications for its involvement in mRNA transport. J Neurosci. 1999; 19:288–297. 10.1523/JNEUROSCI.19-01-00288.1999. 9870958 PMC6782358

[11] Hall MP , Huang S , Black DL . Differentiation-induced colocalization of the KH-type splicing regulatory protein with polypyrimidine tract binding protein and the c-src pre-mRNA. Mol Biol Cell. 2004; 15:774–786. 10.1091/mbc.e03-09-0692. 14657238 PMC329392

[12] Gu W , Pan F , Zhang H , Bassell GJ , Singer RH . A predominantly nuclear protein affecting cytoplasmic localization of β-actin mRNA in fibroblasts and neurons. J Cell Biol. 2002; 156:41–51. 10.1083/jcb.200105133. 11781334 PMC2173579

[13] Shestakova EA , Singer RH , Condeelis J . The physiological significance of beta -actin mRNA localization in determining cell polarity and directional motility. Proc Natl Acad Sci U S A. 2001; 98:7045–7050. 10.1073/pnas.121146098. 11416185 PMC34620

[14] Loreni F , Mancino M , Biffo S . Translation factors and ribosomal proteins control tumor onset and progression: how? Oncogene. 2014; 33:2145–2156. 10.1038/onc.2013.153. 23644661

[15] Liu J , Valencia-Sanchez MA , Hannon GJ , Parker R . MicroRNA-dependent localization of targeted mRNAs to mammalian P-bodies. Nat Cell Biol. 2005; 7:719–723. 10.1038/ncb1274. 15937477 PMC1855297

[16] Teixeira D , Sheth U , Valencia-Sanchez MA , Brengues M , Parker R . Processing bodies require RNA for assembly and contain non-translating mRNAs. RNA. 2005; 11:371–382. 10.1261/rna.7258505. 15703442 PMC1370727

[17] Kedersha N , Anderson P . Mammalian stress granules and processing bodies. Methods Enzymol. 2007; 431:61–81. 10.1016/S0076-6879(07)31005-7. 17923231

[18] Yu JH , Yang WH , Gulick T , Bloch KD , Bloch DB . Ge-1 is a central component of the mammalian cytoplasmic mRNA processing body. RNA. 2005; 11:1795–1802. 10.1261/rna.2142405. 16314453 PMC1370868

[19] Taniuchi K , Furihata M , Hanazaki K , Saito M , Saibara T . IGF2BP3-mediated translation in cell protrusions promotes cell invasiveness and metastasis of pancreatic cancer. Oncotarget. 2014; 5:6832–6845. 10.18632/oncotarget.2257. 25216519 PMC4196166

[20] Taniuchi K , Furihata M , Naganuma S , Saibara T . ARHGEF4 predicts the poor prognosis and promotes cell invasion by influencing ERK1/2 and GSK-3α/β signaling in pancreatic cancer. Int J Oncol. 2018; 53:2224–2240. 10.3892/ijo.2018.4549. 30226582

[21] Taniuchi K , Nishimori I , Hollingsworth MA . Intracellular CD24 inhibits cell invasion by posttranscriptional regulation of BART through interaction with G3BP. Cancer Res. 2011; 71:895–905. 10.1158/0008-5472.CAN-10-2743. 21266361

[22] Taniuchi K , Yawata T , Tsuboi M , Ueba T , Saibara T . Efficient delivery of small interfering RNAs targeting particular mRNAs into pancreatic cancer cells inhibits invasiveness and metastasis of pancreatic tumors. Oncotarget. 2019; 10:2869–2886. 10.18632/oncotarget.26880. 31080558 PMC6499602

[23] Jakymiw A , Pauley KM , Li S , Ikeda K , Lian S , Eystathioy T , Satoh M , Fritzler MJ , Chan EK . The role of GW/P-bodies in RNA processing and silencing. J Cell Sci. 2007; 120:1317–1323. 10.1242/jcs.03429. 17401112

[24] Leary DJ , Terns MP , Huang S . Components of U3 snoRNA-containing complexes shuttle between nuclei and the cytoplasm and differentially localize in nucleoli: implications for assembly and function. Mol Biol Cell. 2004; 15:281–293. 10.1091/mbc.e03-06-0363. 14565981 PMC307547

[25] Holley CL , Li MW , Scruggs BS , Matkovich SJ , Ory DS , Schaffer JE . Cytosolic accumulation of small nucleolar RNAs (snoRNAs) is dynamically regulated by NADPH oxidase. J Biol Chem. 2015; 290:11741–11748. 10.1074/jbc.M115.637413. 25792744 PMC4416874

[26] Bikkavilli RK , Malbon CC . Dishevelled-KSRP complex regulates Wnt signaling through post-transcriptional stabilization of beta-catenin mRNA. J Cell Sci. 2010; 123:1352–1362. 10.1242/jcs.056176. 20332102 PMC2848118

[27] Pai P , Rachagani S , Dhawan P , Batra SK . Mucins and Wnt/β-catenin signaling in gastrointestinal cancers: an unholy nexus. Carcinogenesis. 2016; 37:223–232. 10.1093/carcin/bgw005. 26762229 PMC5014091

[28] Gao S , Bajrami I , Verrill C , Kigozi A , Ouaret D , Aleksic T , Asher R , Han C , Allen P , Bailey D , Feller S , Kashima T , Athanasou N , et al. Dsh homolog DVL3 mediates resistance to IGFIR inhibition by regulating IGF-RAS signaling. Cancer Res. 2014; 74:5866–5877. 10.1158/0008-5472.CAN-14-0806. 25168481

[29] Tanouchi A , Taniuchi K , Furihata M , Naganuma S , Dabanaka K , Kimura M , Watanabe R , Kohsaki T , Shimizu T , Saito M , Hanazaki K , Saibara T . CCDC88A, a prognostic factor for human pancreatic cancers, promotes the motility and invasiveness of pancreatic cancer cells. J Exp Clin Cancer Res. 2016; 35:190. 10.1186/s13046-016-0466-0. 27919290 PMC5139074

[30] Kitamura T , Asai N , Enomoto A , Maeda K , Kato T , Ishida M , Jiang P , Watanabe T , Usukura J , Kondo T , Costantini F , Murohara T , Takahashi M . Regulation of VEGF-mediated angiogenesis by the Akt/PKB substrate Girdin. Nat Cell Biol. 2008; 10:329–337. 10.1038/ncb1695. 18264090

[31] Gherzi R , Lee KY , Briata P , Wegmüller D , Moroni C , Karin M , Chen CY . A KH domain RNA binding protein, KSRP, promotes ARE-directed mRNA turnover by recruiting the degradation machinery. Mol Cell. 2004; 14:571–583. 10.1016/j.molcel.2004.05.002. 15175153

[32] Cammas A , Sanchez BJ , Lian XJ , Dormoy-Raclet V , van der Giessen K , López de Silanes I , Ma J , Wilusz C , Richardson J , Gorospe M , Millevoi S , Giovarelli M , Gherzi R , et al. Destabilization of nucleophosmin mRNA by the HuR/KSRP complex is required for muscle fibre formation. Nat Commun. 2014; 5:4190. 10.1038/ncomms5190. 24969639 PMC4074165

[33] Amirouche A , Tadesse H , Lunde JA , Bélanger G , Côté J , Jasmin BJ . Activation of p38 signaling increases utrophin A expression in skeletal muscle via the RNA-binding protein KSRP and inhibition of AU-rich element-mediated mRNA decay: implications for novel DMD therapeutics. Hum Mol Genet. 2013; 22:3093–3111. 10.1093/hmg/ddt165. 23575223

[34] Giovarelli M , Bucci G , Ramos A , Bordo D , Wilusz CJ , Chen CY , Puppo M , Briata P , Gherzi R . H19 long noncoding RNA controls the mRNA decay promoting function of KSRP. Proc Natl Acad Sci U S A. 2014; 111:E5023–E5028. 10.1073/pnas.1415098111. 25385579 PMC4250097

[35] Connerty P , Bajan S , Remenyi J , Fuller-Pace FV , Hutvagner G . The miRNA biogenesis factors, p72/DDX17 and KHSRP regulate the protein level of Ago2 in human cells. Biochim Biophys Acta. 2016; 1859:1299–1305. 10.1016/j.bbagrm.2016.07.013. 27478153

[36] Michel CI , Holley CL , Scruggs BS , Sidhu R , Brookheart RT , Listenberger LL , Behlke MA , Ory DS , Schaffer JE . Small nucleolar RNAs U32a, U33, and U35a are critical mediators of metabolic stress. Cell Metab. 2011; 14:33–44. 10.1016/j.cmet.2011.04.009. 21723502 PMC3138526

[37] Youssef OA , Safran SA , Nakamura T , Nix DA , Hotamisligil GS , Bass BL . Potential role for snoRNAs in PKR activation during metabolic stress. Proc Natl Acad Sci U S A. 2015; 112:5023–5028. 10.1073/pnas.1424044112. 25848059 PMC4413318

[38] Taniuchi K , Furihata M , Naganuma S , Dabanaka K , Hanazaki K , Saibara T . Podocalyxin-like protein, linked to poor prognosis of pancreatic cancers, promotes cell invasion by binding to gelsolin. Cancer Sci. 2016; 107:1430–1442. 10.1111/cas.13018. 27461278 PMC5084665

[39] Novichkova S , Egorov S , Daraselia N . MedScan, a natural language processing engine for MEDLINE abstracts. Bioinformatics. 2003; 19:1699–1706. 10.1093/bioinformatics/btg207. 12967967

[40] Taylor AM , Dieterich DC , Ito HT , Kim SA , Schuman EM . Microfluidic local perfusion chambers for the visualization and manipulation of synapses. Neuron. 2010; 66:57–68. 10.1016/j.neuron.2010.03.022. 20399729 PMC2879052

[41] Li TS , Yawata T , Honke K . Efficient siRNA delivery and tumor accumulation mediated by ionically cross-linked folic acid-poly(ethylene glycol)-chitosan oligosaccharide lactate nanoparticles: for the potential targeted ovarian cancer gene therapy. Eur J Pharm Sci. 2014; 52:48–61. 10.1016/j.ejps.2013.10.011. 24178005

