# Correction: KHSRP-bound small nucleolar RNAs associate with promotion of cell invasiveness and metastasis of pancreatic cancer

**DOI:** 10.18632/oncotarget.28361

**Published:** 2023-02-07

**Authors:** Keisuke Taniuchi, Mitsunari Ogasawara

**Affiliations:** ^1^Department of Gastroenterology and Hepatology, Kochi Medical School, Kochi University, Nankoku, Kochi 783-8505, Japan; ^2^Department of Endoscopic Diagnostics and Therapeutics, Kochi Medical School, Kochi University, Nankoku, Kochi 783-8505, Japan

**This article has been corrected:** In Figure 9, the panel C image contains an accidental partial overlap of the panel D image. The corrected Figure 9, produced using the original data, is shown below. The authors declare that these corrections do not change the results or conclusions of this paper.

Original article: Oncotarget. 2020; 11:131–147. 131-147. https://doi.org/10.18632/oncotarget.27413

**Figure 9:**
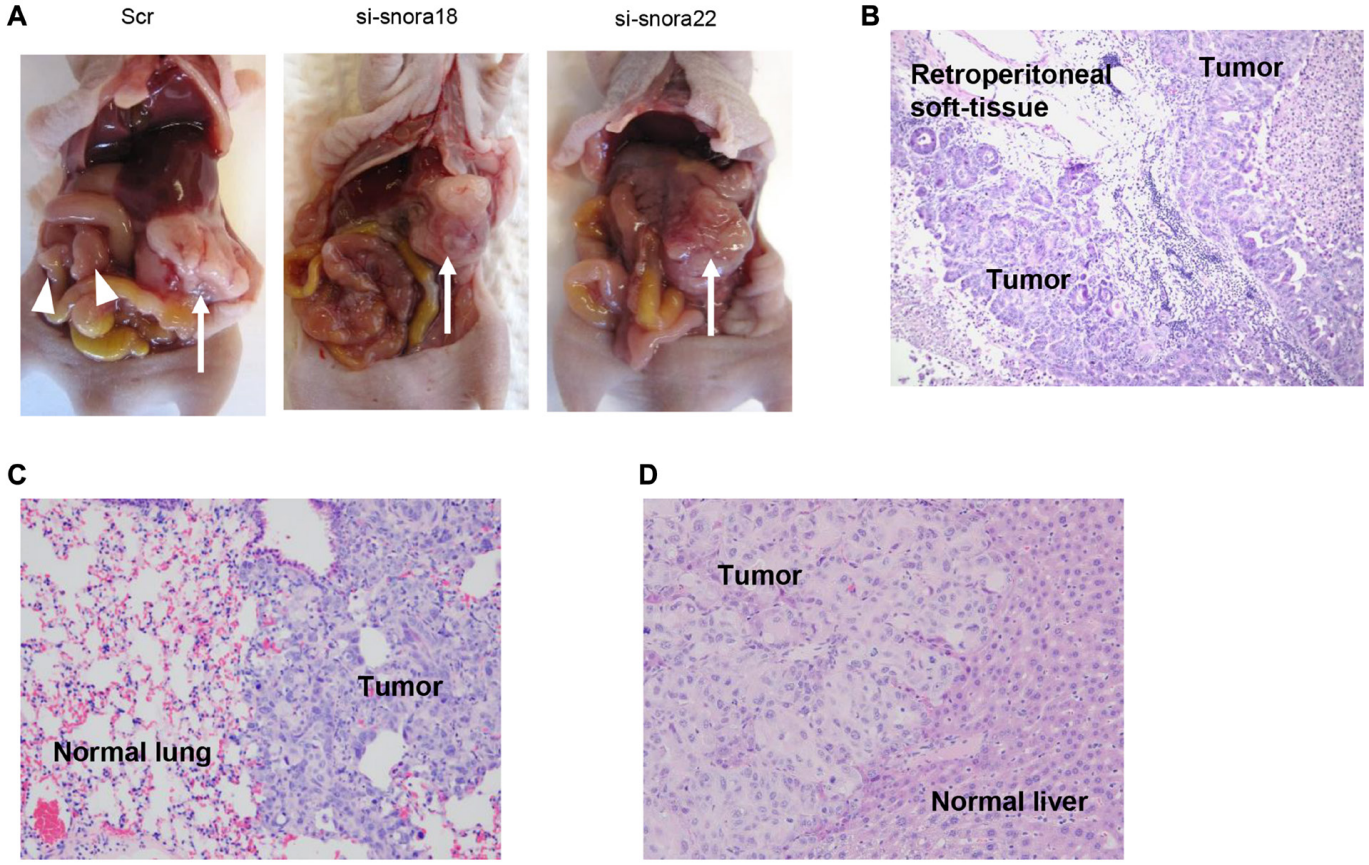
Knockdown effect of siRNA-FA-PEG-COL nanoparticles targeting KHSRP-bound snoRNAs on cell motility and invasion in the orthotopic murine model of PDAC. (**A**) Development of carcinomatosis in S2-013 tumor-bearing mice treated with scrambled control siRNA-FA-PEG-COL nanoparticles (Scr) and target siRNA-FA-PEG-COL nanoparticles against *SNORA18* (si-snora18) and *SNORA22* (si-snora22). Arrow, primary tumor; arrowheads, dissemination nodules in the abdominal cavity. (**B**–**D**) Hematoxylin and eosin staining of representative sections of S2-013-derived PDAC tumor tissues in mice treated with scrambled control siRNA-FA-PEG-COL nanoparticles showing areas of regional invasion of the retroperitoneum (B) and distant metastases to the lung (C) and liver (D). Original magnification: 200×.

