# Supplementary material for: KHSRP-bound small nucleolar RNAs associate with promotion of cell invasiveness and metastasis of pancreatic cancer
Source: Oncotarget. 2020 Jan 14;11(2):131–47. doi: 10.18632/oncotarget.27413 (PMC6968780; doi:10.18632/oncotarget.27413)
Supplement: Supplementary file 1 [file oncotarget-11-131-s001.pdf]

KHSRP-bound small nucleolar RNAs associate with promotion of cell invasiveness and metastasis of pancreatic cancer

SUPPLEMENTARY MATERIALS

Supplementary Table 1: RNAs that co-immunoprecipitate with KHSRP. See Supplementary Table 1

Supplementary Table 2: TOP10 GO terms of KHSRP-bound RNAs

1. Biological Process

Accession	GO Term	*P*-value
GO:0006915	apoptosis	1.45E-13
GO:0008380	RNA splicing	4.73E-13
GO:0006412	translation	1.02E-11
GO:0010467	gene expression	4.02E-11
GO:0000398	nuclear mRNA splicing, via spliceosome	5.99E-11
GO:0006366	transcription from RNA polymerase II promoter	7.90E-10
GO:0006281	DNA repair	1.07E-09
GO:0000278	mitotic cell cycle	1.28E-08
GO:0008633	activation of pro-apoptotic gene products	3.90E-08
GO:0006397	mRNA processing	6.55E-08

2. Cellular Component

Accession	GO Term	*P*-value
GO:0005634	nucleus	1.65E-55
GO:0005737	cytoplasm	2.52E-44
GO:0005739	mitochondrion	3.01E-26
GO:0005829	cytosol	1.74E-24
GO:0005730	nucleolus	2.06E-24
GO:0005654	nucleoplasm	9.30E-16
GO:0005840	ribosome	2.05E-10
GO:0005622	intracellular	3.62E-08
GO:0005743	mitochondrial inner membrane	4.39E-08
GO:0005694	chromosome	2.12E-07

3. Molecular Function

Accession	GO Term	*P*-value
GO:0005515	protein binding	5.27E-59
GO:0003723	RNA binding	2.71E-15
GO:0000166	nucleotide binding	6.29E-13
GO:0003735	structural constituent of ribosome	2.95E-10
GO:0046872	metal ion binding	1.43E-09
GO:0008270	zinc ion binding	6.71E-08
GO:0003677	DNA binding	1.37E-07
GO:0005524	ATP binding	8.17E-07
GO:0016740	transferase activity	1.49E-06
GO:0000049	tRNA binding	6.98E-06
